# Multi-Elemental Profiling of Tibial and Maxillary Trabecular Bone in Ovariectomised Rats

**DOI:** 10.3390/ijms17060977

**Published:** 2016-06-21

**Authors:** Pingping Han, Shifeier Lu, Yinghong Zhou, Karine Moromizato, Zhibin Du, Thor Friis, Yin Xiao

**Affiliations:** 1 Institute of Health and Biomedical innovation, Queensland University of Technology, Brisbane 4059, Australia; p.han@uq.edu.au (P.H.); shifeier.lu@connect.qut.edu.au (S.L.); zhibin.du@qut.edu.au (Z.D.); t.friis@qut.edu.au (T.F.); 2 Tissue Engineering and Microfluidics Laboratory, Australian Institute for Bioengineering and Nanotechnology, University of Queensland, St. Lucia 4072, Australia; 3 Institute for Future Environments, Queensland University of Technology, Brisbane 4000, Australia; k.harumimoromizato@qut.edu.au

**Keywords:** ovariectomised rats, tibia, maxilla, LA-ICP-MS, osteoporosis

## Abstract

Atomic minerals are the smallest components of bone and the content of Ca, being the most abundant mineral in bone, correlates strongly with the risk of osteoporosis. Postmenopausal women have a far greater risk of suffering from OP due to low Ca concentrations in their bones and this is associated with low bone mass and higher bone fracture rates. However, bone strength is determined not only by Ca level, but also a number of metallic and non-metallic elements in bone. Thus, in this study, the difference of metallic and non-metallic elements in ovariectomy-induced osteoporosis tibial and maxillary trabecular bone was investigated in comparison with sham operated normal bone by laser ablation inductively-coupled plasma mass spectrometry using a rat model. The results demonstrated that the average concentrations of ^25^Mg, ^28^Si, ^39^K, ^47^Ti, ^56^Fe, ^59^Co, ^77^Se, ^88^Sr, ^137^Ba, and ^208^Pb were generally higher in tibia than those in maxilla. Compared with the sham group, Ovariectomy induced more significant changes of these elements in tibia than maxilla, indicating tibial trabecular bones are more sensitive to changes of circulating estrogen. In addition, the concentrations of ^28^Si, ^77^Se, ^208^Pb, and Ca/P ratios were higher in tibia and maxilla in ovariectomised rats than those in normal bone at all time-points. The present study indicates that ovariectomy could significantly impact the element distribution and concentrations between tibia and maxilla.

## 1. Introduction

Osteoporosis (OP) is a progressive systemic skeletal disease affecting 200 million people worldwide [1]. It is characterized by low bone mass and inferior strength that leads to increased fragility and risk of bone fractures. Clinical research has shown there is an inverse relationship between serum estrogen levels and the healing capacity of fractured long and axial bones that affects both women and men [2,3,4,5]. In addition, estrogen deficiency may lead to jawbone resorption and delayed alveolar bone wound healing in osteoporotic patients [6]. Ovariectomy (Ovx) induces “postmenopausal” OP in rats and is a commonly-used experimental model to better understand the pathophysiological mechanisms of OP. This model is characterized by continuous bone loss and an increased rate of bone turnover due to estrogen deficiency [7,8,9].

It has been observed that the impact of experimental Ovx on bone loss is site-dependent and that the severity in bones varies according to different size, mass and density [10]. Further evidence has demonstrated that trabecular bones are more sensitive to estrogen deficiency than other skeletal sites, including porous maxillary bones [11]. Recent research has confirmed that Ovx leads to a significant reduction of bone mass in femurs and tibia, which are formed by endochondral ossification, but not in maxilla, which is formed by intramembranous ossification [12]. Most studies have tended to focus on the bone loss in long bone and mandibular alveolar bone after Ovx, using three-dimensional (3D) micro-computed tomography (µCT) [8], dual-energy X-ray absorptiometry (DEXA) [13], and Raman spectroscopy [14]; however, the effects of Ovx on the bone remodelling in maxilla and tibia remain unclear.

It is well documented that organic and inorganic components both contribute to bone toughness and rigidity. Clinical research has shown a strong correlation between OP and low Ca concentrations in the bones of postmenopausal women which is associated with low bone mass and high bone fracture rates [15,16]. This association has been confirmed in Ovx rats, where there is a gradual and significant increase in serum Ca and P when compared to controls [17,18]. Moreover, there are a number of reports that highlight the importance of atomic composition in bone. Environmental and occupational exposure to cadmium (Cd) is linked to a significant decrease in bone mineral density, particularly in men [19]. It is thought that Cd interferes with the renal enzymes that hydroxylate calcidiol to calcitriol [20]. In a cohort of postmenopausal Indonesian women it was found that the concentrations of B, Al, S, V, Co, Mo, Te, Ba, La, Ni, As, and Ca/P ratio were higher in those with OP compared to age-matched controls with normal bone density [21]. Most of the previous literature has focused on the relationship between environmental exposure to metals and bone mineral quality [22], whereas the association between atomic minerals and bone remodelling at multiple skeletal sites has not yet been assessed.

There are no reported studies that detail the differences of atomic mineral distribution in maxilla and tibia of Ovx rats in a time-dependent manner. We hypothesized that there would be a significant difference in the content of bone mineral elements between maxillary alveolar bone and tibia (long bone) of Ovx rats over time. We tested this hypothesis using laser ablation inductively-coupled plasma mass spectrometry (LA-ICP-MS). This is a powerful analytical tool for the quantification of trace element concentrations, and has a spatial resolution ranging from 10 to 100 μm and a detection limit ranging from parts per million to parts per billion [23,24].

The objective of this study was, therefore, to investigate the characteristics of atomic mineral changes in maxillary and tibial bones arising from Ovx in female rats. LA-ICP-MS was applied to compare the changes in various skeletal sites at defined time points over a period of 20 weeks post-surgery. A total of 27 elements (B, Al, S, V, Co, Mo, Te, Ba, La, Ni, As, Na, Mg, P, K, Ca, Cr, Pd, Ag, Mn, Fe, Cu, Zn, Rb, Sr, Pb, and Se) were analysed. The present study provides high resolution of the atomic mineral compositions of bone in general and, more specifically, gives us a better understanding of the association between atomic mineral composition and OP.

## 2. Results

### 2.1. The Effect of OVX on the Average Concentrations of Atomic Minerals in Tibia and Maxilla at Defined Time Points

The average concentrations of ^25^Mg, ^28^Si, ^39^K, ^47^Ti, ^56^Fe, ^59^Co, ^77^Se, ^88^Sr, ^137^Ba, and ^208^Pb were analysed by LA-ICP-MS in both tibia and maxilla at set time points (Table 1). In general, Ovx rats had higher concentrations of all the evaluated elements in the tibia compared to maxilla at the individual time points. In the tibia, the concentrations of ^28^Si, ^39^K, ^77^Se, and ^208^Pb were greater in osteoporotic bones compared to the controls (Table 1; *, *p* < 0.05). Likewise, maxillary bones of Ovx rats had greater concentrations of ^28^Si, ^77^Se, ^88^Sr, ^137^Ba, and ^208^Pb compared to controls (Table 1; *, *p* < 0.05). It was also noteworthy that Ovx resulted in greater concentrations of ^28^Si, ^77^Se, and ^208^Pb in both tibia and maxilla compared with the sham group over the time course.

### 2.2. Ca/P Ratios of Tibia and Maxilla

**Figure 1:**
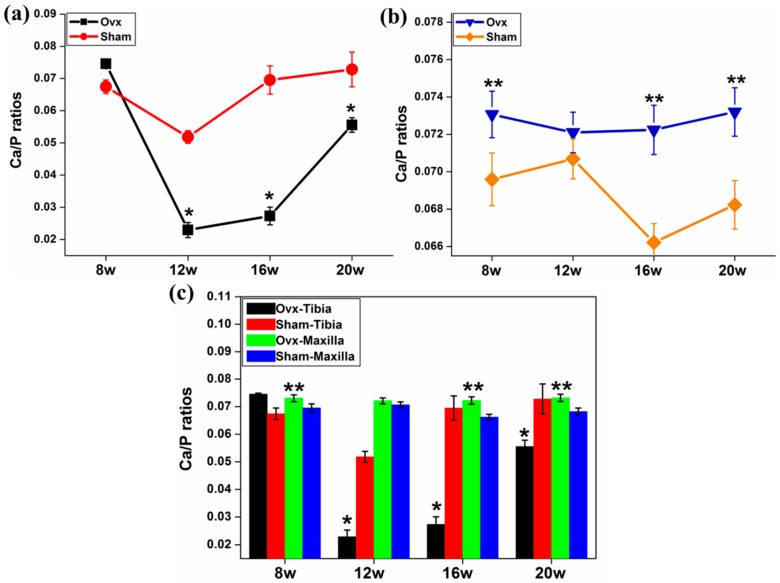
Ca/P ratios in tibia and maxilla. (**a**) The Ca/P ratios decreased dramatically in the Ovx-tibia at 12 weeks but recovered towards week 20; (**b**) There was an increasing trend of Ca/P ratios in the Ovx-maxilla compared to the samples from the sham group; (**c**) Tibial bones from the Ovx rats had lower Ca/P ratios compared to the maxilla from the same animals. The Ca/P ratios remained unchanged in the Ovx-maxilla (the ratios were normalised against matrix-matched reference materials). *: Significant difference (*p* < 0.05) between Ovx-tibia and Sham-tibia; **: Significant difference (*p* < 0.05) between Ovx-maxilla and Sham-maxilla.

LA-ICP-MS was also used to determine if there were discernible changes to Ca and P concentrations in the tibia and maxilla of Ovx- *versus* sham-operated rats. The trend was towards an increase of the Ca/P ratio in Ovx animals in both tibia and maxilla over the time course compared to the sham group at 12, 16, and 20 weeks after the surgery (Figure 1a,b). Tibial trabecular bones from the Ovx rats had lower Ca/P ratio values compared to the maxilla from the same animals (Figure 1c).

### 2.3. The Concentrations of Toxic Atomic Minerals, ^*59*^Co and ^*208*^Pb, in Tibia and Maxilla

**Figure 2:**
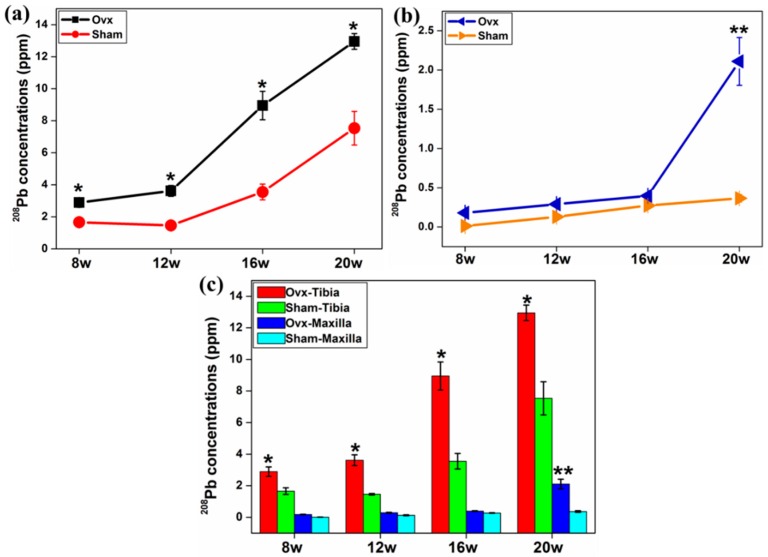
Ovx increased the average concentrations of ^208^Pb (normalised against matrix-matched reference materials) for both tibia (**a**) and maxilla (**b**) at all time points post-surgery, compared to the sham group; and (**c**) tibial bone accumulated more ^208^Pb compared to maxillary bone. *: Significant difference (*p* < 0.05) between Ovx-tibia and Sham-tibia; **: Significant difference (*p* < 0.05) between Ovx-maxilla and Sham-maxilla.

**Figure 3:**
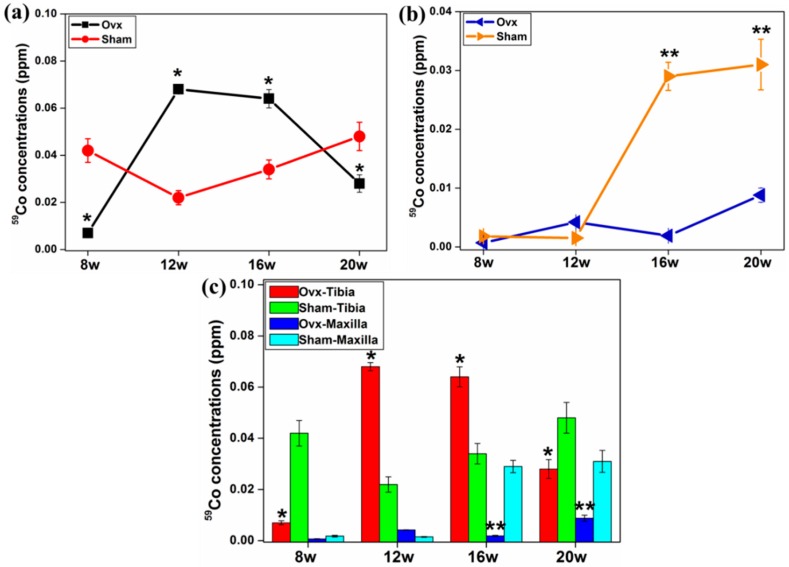
The average concentrations of ^59^Co in tibia (**a**) and maxilla (**b**) at different time points post-surgery. (**a**,**b**) At 16 week, there was a higher concentration of ^59^Co in both tibia and maxilla in Ovx rats compared to the sham-operated rats; and (**c**) comparison of ^59^Co concentrations in tibia and maxilla over a time course post-surgery. *: Significant difference (*p* < 0.05) between Ovx-tibia and Sham-tibia; **: Significant difference (*p* < 0.05) between Ovx-maxilla and Sham-maxilla.

The average concentrations of ^59^Co and ^208^Pb in tibia and maxilla were evaluated in this study. The concentrations of ^208^Pb were higher in both tibia and maxilla from the Ovx group than the sham group over the time course (Figure 2a,b). More importantly, the concentration of ^208^Pb in tibia was greater than that in the maxilla at all time points (Figure 2c). Higher concentrations of ^59^Co were detected in tibial bones of the Ovx rats at both 12 and 16 weeks compared to the controls (Figure 3a). Furthermore, there was a significantly greater concentration of ^59^Co in the maxilla at both 16 and 20 weeks in the Ovx group compared to the controls (Figure 3b), and the tibia of the Ovx animals had a higher concentration of ^59^Co than the maxilla from the same animals over all time points (Figure 3c).

### 2.4. The Effect of Ovx on the Concentrations of ^*66*^Zn, ^*88*^Sr, and ^*137*^Ba in Tibia and Maxilla

**Figure 4:**
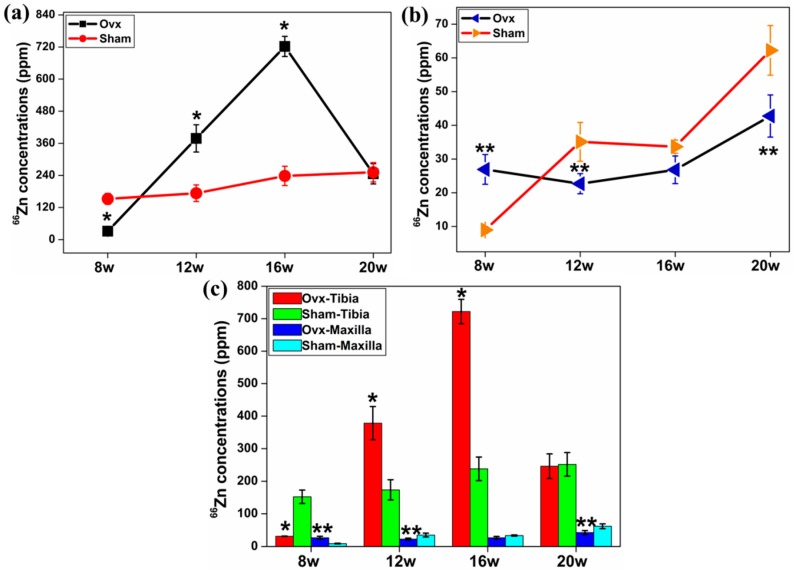
The average concentrations of ^66^Zn in both tibia (**a**) and maxilla (**b**) after normalisation at different time points post-surgery. (**a**) Ovx resulted in higher concentration of ^66^Zn in tibia at 12 and 16 weeks; (**b**) there was a higher concentration of ^66^Zn in maxilla from the Ovx rats at eight weeks, while the sham group had a higher ^66^Zn concentration at the other time points; and (**c**) the tibia had distinctly higher concentration of ^66^Zn compared to the maxilla. *: Significant difference (*p* < 0.05) between Ovx-tibia and Sham-tibia; **: Significant difference (*p* < 0.05) between Ovx-maxilla and Sham-maxilla.

Ovx resulted in higher levels of ^66^Zn in tibia at 12 and 16 weeks compared to the controls (Figure 4a). There was a higher concentration of ^66^Zn in the maxilla of the Ovx group at week eight, whereas the maxilla of the control group had higher levels of ^66^Zn at the other time points (Figure 4b). In both groups tibia had higher levels of ^66^Zn compared to the maxilla at each time point (Figure 4c).

**Figure 5:**
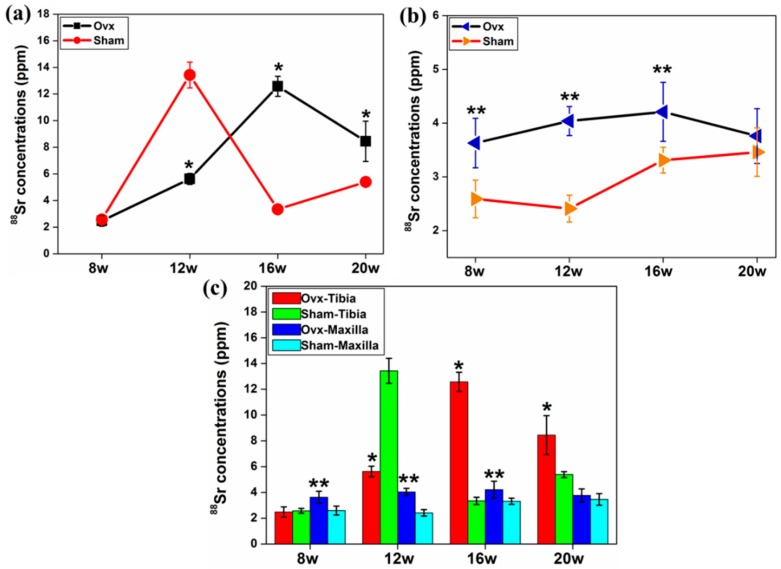
(**a**) The average concentrations of ^88^Sr increased in Ovx tibia at weeks 16 and 20 post surgery; (**b**) the concentration of ^88^Sr was higher in Ovx maxilla at all time points; and (**c**) generally, there was a higher concentration of ^88^Sr in the maxilla from the Ovx group compared to the sham group at all time points post-surgery. *: Significant difference (*p* < 0.05) between Ovx-tibia and Sham-tibia; **: Significant difference (*p* < 0.05) between Ovx-maxilla and Sham-maxilla.

The average concentration of ^88^Sr in the tibia of the Ovx group was greater at 16 and 20 weeks after surgery compared to the controls (Figure 5a). The concentration of ^88^Sr was higher in maxillary bones in Ovx rats at all time points compared to the sham group (Figure 5b,c).

**Figure 6:**
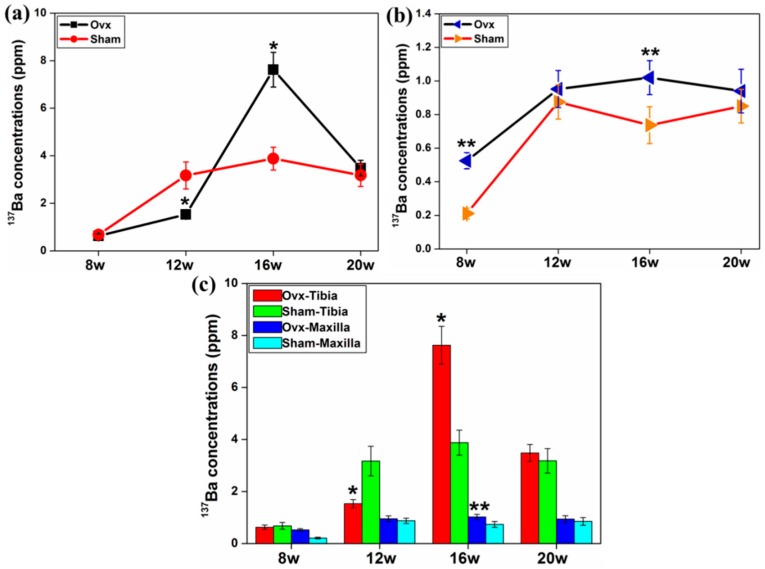
(**a**) The effect of Ovx on the concentrations of ^137^Ba in tibia and (**b**) maxilla at different time points post-surgery. Ovx increased the concentration of ^137^Ba in maxilla across all the time points; and (**c**) higher concentration of ^137^Ba was detected in tibia than in maxilla, peaking at week 16. *: Significant difference (*p* < 0.05) between Ovx-tibia and Sham-tibia; **: Significant difference (*p* < 0.05) between Ovx-maxilla and Sham-maxilla.

The concentration of ^137^Ba was greater in the maxilla of the Ovx group at all time points compared with controls (Figure 6b) and in both groups the concentration of ^137^Ba was greater in tibia than in maxilla at all time points (Figure 6c).

## 3. Discussion

The relationship that exists between OP and the site-dependent distribution of atomic minerals in bone still remains unclear and is especially the case for the tibia and maxilla. In this study, Ovx rats were used as a model to mimic the bone loss characteristic of human OP caused by estrogen deficiency [25]. This is the first study of its kind to investigate the effects of Ovx-induced estrogen deficiency on the distribution of atomic mineral elements in tibial and maxillary trabecular bone using LA-ICP-MS, with the aim of mapping the site-specific chemical compositions. These results demonstrated that the concentrations of ^25^Mg, ^28^Si, ^39^K, ^47^Ti, ^56^Fe, ^59^Co, ^77^Se, ^88^Sr, ^137^Ba, and ^208^Pb were, on average, greater in the tibia than in the maxilla, suggesting that tibial trabecular bones are more sensitive to changes in estrogen levels than maxillary trabecular bones. We found that the concentrations of ^28^Si, ^77^Se, ^208^Pb, and Ca/P ratios were higher in osteoporotic tibia and maxilla compared to controls at all time-points. This suggests that these minerals may have a negative effect on the balance between bone resorption and bone formation activity.

In order to establish how Ovx affect bone quality, researchers have tended to rely on the conventional approaches, such as histomorphometry [8,26], µCT [8,27], DEXA [28] and Raman spectroscopy [14]. These methods do, however, fail to reveal the distributions of the inorganic and organic elements of bone. In this study, we employed LA-ICP-MS to determine the roles of key atomic minerals in OP. LA-ICP-MS has a spatial resolution ranging from 10 to 100 µm [23] and a detection range as low as parts per billion [24]. These properties make LA-ICP-MS a powerful tool with which to quantify the elements of hard tissues and allow us to get a clearer picture of the bone mineralization processes [29,30,31]. In this study, bovine bone pellets were used as matrix-matched reference materials since such pellets have a consistent element distribution that makes them a homogenous reference material. NIST 610 glass wafers were used as an internal standard.

The ratio of Ca to P in bone has been used as an indicator for OP. Increased Ca intake has been shown to increase bone density [32], whereas consuming excess amounts of dietary P combined with a low Ca intake, leads to secondary hyperparathyroidism and progressive decrease in bone mineral contents [33]. In addition, a decreased Ca/P ratio is associated with increasing bone turnover [34]. It still remains controversial whether Ovx-induced estrogen deficiency has a direct effect on Ca/P ratios. One study, which analysed differences in the levels of Ca, P, Fe, Cu, Zn, Ni, Ca/P, and Cu/Zn between Ovx and controls at four and eight weeks post-surgery using X-ray fluorescence, found no significant difference in these parameters [35]. However, another study showed a gradual but significant increase in serum Ca and P level in Ovx rats [18]. In the present study, we applied LA-ICP-MS to investigate the concentration of elements in resin-embedded tibia and maxilla with polished smooth surfaces. Significantly, we found that Ovx-induced estrogen deficiency led to an increase in the Ca/P ratios in both tibia and maxilla compared with the sham-operated rats in a time-dependent manner.

Up to 94% of the body burden of Pb is found in organic or inorganic forms in bone and have half-lives spanning from years to decades [36]. Pb is reported to inhibit Ca absorption and cellular function, and can induce OP by inhibiting the function of vitamin D [37]. Pb has been shown *in vitro* to interfere with the functioning of the Ca binding protein osteonectin in osteoblasts-like ROS 17/2.8 cells [36]. We found that the concentration of ^208^Pb was significantly higher in both tibia and maxilla in the Ovx group compared to the sham group over time. Interestingly, the concentration of ^208^Pb was greater in tibia compared to the maxilla at every time point. A number of recent studies show that an accumulation of Pb in bone disturbs cellular functions, which leads to the imbalance of bone resorption and bone formation that is characteristic of OP [38,39,40].

We measured ^59^Co at higher concentrations in the tibia (Figure 4a) and maxilla of the Ovx group compared to the controls at both 16 and 20 weeks (Figure 3b). The toxic effect of Co has been known since the 1970s when clinicians began reporting complications associated with cobalt-containing prosthetic devices [41,42]. Co has been shown to affect the redox state in the osteoblasts-like cell line MG-63, which leads to increased protein oxidation [43,44]. In the osteosarcoma cell line Saos2, Coexposure reduces the ratio of osteoprotegerin (OPG) to receptor activator of nuclear factor kappa B ligand (RANKL) [45]. OPG is a decoy receptor for RANKL and *in vivo* the ratio between the two determines the activation and regulation of osteoclastogenesis [46]. In the present study, the higher level of ^59^Co found in the Ovx group is likely to have led to increased osteoclast activity and contributed to the imbalance between bone formation and resorption activity that was apparent by the induced OP in these animals.

Zn and Sr are both essential elements for normal bone growth and metabolism. Zn, for example, is incorporated in all six classes of metalloenzymes, including the important bone enzymes alkaline phosphatase, whereas the tartrate-resistant acid phosphatase (TRAP) enzyme is inhibited by Zn [47]. Osteoporotic patients have significantly lower serum Zn concentrations and higher urine Zn concentrations compared to osteoarthritic patients. The latter could be due to higher bone resorption but it may also be due to lower renal Zn reabsorption which would result in an overall loss of Zn [48]. Our study showed evidence of a Zn deficiency in the maxillary bone of the Ovx rats and suggests a causal relationship with the osteoporotic state of these animals. Sr has garnered considerable attention as an anti-OP agent since it was discovered to have potent inhibitory effects on bone resorption [49]. In a study similar to ours, Ovx rats, when treated with the Sr salt S12911, maintained a bone dry and ash weight similar to sham controls. Other measures of bone formation, such as plasma alkaline phosphatase (ALP) and osteocalcin, were also elevated or even increased in Ovx rats following S12911 treatment [50]. In the present study, the level of Sr in osteoporotic tibial bone was generally higher than in the osteoporotic maxillary bone. However, there was higher concentration of Sr detected in the maxilla from the Ovx group compared with the sham group at all time points post-surgery. This difference in the distribution between tibia and maxilla needs further investigation.

Ba is a divalent metal with properties similar to Ca and has no known biological role. In general, Ba^2+^ ions are toxic or inhibitory to cellular processes and in humans the lethal dose of BaCl_2_ is 800–900 mg. The chemical property of Ba allows it to readily compete with and replace Ca in bone, which may lead to OP. Environmental Ba in water or animal food can impair Ca metabolism [51] and it has been estimated that there is a ten-fold skeletal accumulation of Ba from the second to the eighth decade of life in humans [52]. Ba titanate is a piezoelectric ceramic that has been shown to promote osteogenesis and which, paradoxically, appears to have good tissue compatibility [53]. Bone is naturally a piezoelectric material which is caused by collagen fibres slipping past one another. This creates electrical dipoles that attract Ca^2+^ and PO_4_^3−^ to opposite electrical charges and which stimulate bone growth. The piezoelectric coefficient of hydroxy-apatite/barium titanate (HA/BT) are comparable with those of cancellous bone and appears to have no cytotoxic effects [54]. In our study we found a greater amount of Ba in osteoporotic bone compared to the controls and further demonstrated that there was a higher concentration of ^137^Ba detected in tibia than in maxilla in every time point. In the context of this study, this suggests that Ba was involved in the imbalance between bone formation and bone resorption in the Ovx animals.

## 4. Materials and Methods

### 4.1. Animals

Sprague-Dawley female rats (three months old, *n* = 48) were used in the study, which had the approval of the Animal Ethics Committees of Queensland University of Technology and Griffith University. One group of rats underwent sham operations (Sham, *n* = 24) whereas the other group underwent bilateral Ovx, *n* = 24, as described previously [8,55]. Briefly, ligatures were placed at the end of fallopian tube to exteriorize the ovaries of the Ovx group. The sham operation consisted of removing fat tissue near the ovaries of approximately the same size. At weeks eight, twelve, sixteen, and twenty post-surgery, six rats from each group were sacrificed with an overdose of ketamine. Tibial and maxillary bones were harvested from each animal and fixed in 4% paraformaldehyde (PFA, Sigma-Aldrich, Castle Hill, NSW, Australia) for 24 h. The specimens were dehydrated in a series of graded alcohols and embedded in an acrylic resin (Technovit 7200, Heraeus Kulzer GmbH, Wehrheim, Germany).

### 4.2. LA-ICP-MS Analysis for Tibia and Maxilla Bones

**Figure 7:**
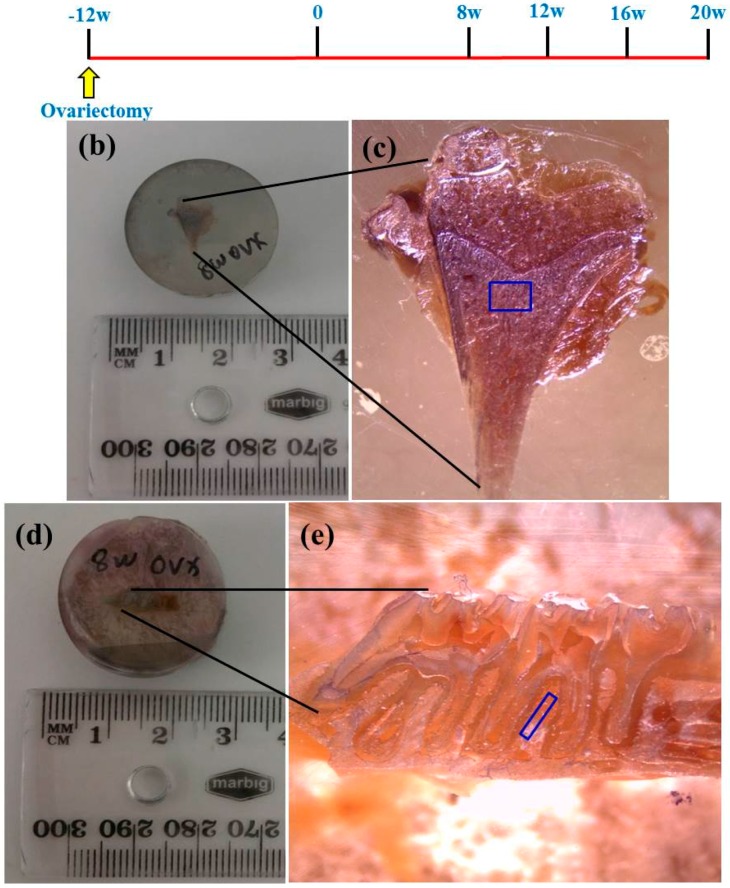
(**a**) The rats underwent either Ovx or sham operation at three months of age and were sacrificed at eight, twelve, sixteen or twenty weeks post-surgery. Note: *n* = 6 for each group at each time point; (**b**,**d**) Actual size of the resin-embedded tibia and maxilla; and (**c**,**e**) areas of interest that were probed for element concentrations.

The atomic element distributions in the resin-embedded tibia and maxilla were analysed using the glass matrices NIST SRM 612 and 610 (National Institute of Standards and Technology, Gaithersburg, MD, USA) as external calibration standards. In this study, we used bovine bone pellets as matrix-matched reference materials to further calibrate the element concentrations, as described previously [56]. For LA-ICP-MS, we used an Agilent 8800 single collector, quadrupole ICP-MS (Agilent Technologies Inc., Santa Clara, CA, USA) with a 193 nm wavelength excimer laser and 2-volume Trueline ablation cell from ESI New Wave Research (Bozeman, MT, USA). Parameters, such as gas flows and repetition rate, were optimized for laser ablation analysis. The resin samples and NIST standards were secured in the laser sample chamber, in which Helium (He) was used as a carrier gas and was repeatedly evacuated and back-filled with He to eliminate changes to the gas composition (air + He) from the introduction system. The laser was set to a modest energy output generating a fluence of 3.0 J/cm^2^, and a pulse rate of 10 Hz. A masked rectangular beam of 15 × 150 µm was moved parallel to the short dimension at 5 µm/s to create a track approximately 750 µm long and 15 µm deep in the tissues as shown in Figure 7. ICP-MS operating parameters were summarized in Table 2. The time to cycle through the nine selected isotopes was 0.12 s. Scans of varying duration were bracketed by 50 s of background (laser-off) data acquisition. Data were analysed and displayed using IOLITE [57].

After the samples were secured in the ablation chamber, the whole system was purged with He carrier gas at 0.5 L·min^−1^ for 3 h prior to each measurement to minimise noise from the ^13^C background. In addition, a background signal was collected during the first 30 s of analysis.

### 4.3. Data Analysis

The data were processed using the SPSS software (SPSS Inc., Chicago, IL, USA). The element concentrations determined for each sample were displayed with mean ± SD (standard deviation) values.

## 5. Conclusions

In summary, we applied LA-ICP-MS to investigate the profile of key atomic mineral elements in osteoporotic rat bones and unveiled changes to the concentrations of mineral elements in both tibial and maxillary bones in a site-specific and time-dependent manner. Our findings provide a fundamental overview of metallic and non-metallic elements in Ovx-induced osteoporotic bone which will help the understanding of bone strength.

## Figures and Tables

**Table 1:** The effect of Ovx on the average concentrations of atomic minerals on tibia and maxilla bones with time-course (* *p* < 0.05).

Element	Concentration						Time Points
	NIST 610	Bone Pellets	Tibia		Maxilla		
			Ovx (*n* = 6)	Sham (*n* = 6)	Ovx (*n* = 6)	Sham (*n* = 6)	
Mg 24	464.9 ± 5.36	155.88 ± 3.38	283 ± 23.53	1335 ± 199.42	166.57 ± 3.10 *	159.33 ± 2.19	8 weeks
			2513 ± 91.19 *	1697.5 ± 210.78	174.37 ± 5.69	228.2 ± 5.36	12weeks
			2810 ± 310 *	1115 ± 167.63	164.5 ± 9.19	174.86 ± 8.14	16 weeks
			1794.5 ± 255.2 *	1310 ± 113.58	540 ± 78.1 *	358.4 ± 36.12	20 weeks
Si 28	326,975 ± 1027	3.03 ± 0.31	1410 ± 200.74 *	355.67 ± 23.54	2.35 ± 0.31	5.22 ± 0.41	8 weeks
			1283.3 ± 137.8 *	473.33 ± 45.83	8.9 ± 1.04 *	2.77 ± 0.25	12weeks
			1545 ± 164.15 *	440 ± 24.13	21.29 ± 3.54 *	2.34 ± 0.31	16 weeks
			2670 ± 188.91 *	430 ± 42.43	45.15 ± 5.28 *	2.83 ± 0.29	20 weeks
K 39	487 ± 17.11	4.94 ± 0.34	62.09 ± 5.21	96.25 ± 17.31	9.48 ± 1.08 *	5.91 ± 0.56	8 weeks
			968 ± 109.03 *	126.33 ± 15.45	26.14 ± 2.52	34.13 ± 4.07	12weeks
			272.33 ± 25.65 *	170.25 ± 20.33	35.23 ± 5.37 *	25.95 ± 2.74	16 weeks
			622 ± 50.91 *	237.5 ± 17.61	55.5 ± 7.06 *	42.2 ± 5.21	20 weeks
Ti 47	433.9 ± 1.53	0.45 ± 0.07	1.67 ± 0.18 *	0.77 ± 0.11	0.22 ± 0.02	0.30 ± 0.01	8 weeks
			2.37 ± 0.31 *	0.62 ± 0.05	0.39 ± 0.03	0.31 ± 0.04	12weeks
			3.53 ± 0.56	5.01 ± 0.45	0.29 ± 0.001	0.36 ± 0.02	16 weeks
			2.1 ± 0.32	5.3 ± 0.51	0.45 ± 0.05	0.41 ± 0.05	20 weeks
Fe 56	456.4 ± 8.13	1.89 ± 0.23	31.05 ± 5.01	424.33 ± 10.26	1.86 ± 0.23	1.81 ± 0.25	8 weeks
			1594 ± 152 *	464 ± 26.9	2.03 ± 0.15	2.46 ± 0.31	12weeks
			1554 ± 152.1 *	623.33 ± 90.73	2.8 ± 0.63	2.78 ± 0.42	16 weeks
			839.5 ± 55.9	1015 ± 91.9	3.86 ± 0.35	3.81 ± 0.34	20 weeks
Co 59	405.1 ± 6.12	0.12 ± 0.006	0.007 ± 0.0008	0.042 ± 0.005	0.0007 ± 0.0001	0.0018 ± 0.00035	8 weeks
			0.068 ± 0.0016 *	0.022 ± 0.003	0.0042 ± 0.0001 *	0.0015 ± 0.00016	12weeks
			0.064 ± 0.0039 *	0.034 ± 0.004	0.0019 ± 0.0002	0.029 ± 0.0024	16 weeks
			0.028 ± 0.0037	0.048 ± 0.006	0.0088 ± 0.0012	0.031 ± 0.0043	20 weeks
Zn 66	456 ± 5.56	3.52 ± 0.26	31.4 ± 0.71	152.2 ± 20.61	26.92 ± 4.41	8.96 ± 1.03	8 weeks
			378.25 ± 51.02 *	173.25 ± 31.01	22.68 ± 2.98	35.1 ± 5.76	12weeks
			722 ± 37.61 *	238 ± 36.13	26.81 ± 4.09	33.65 ± 1.93	16 weeks
			246 ± 37.98	251.67 ± 36.16	42.75 ± 6.25	62.21 ± 7.35	20 weeks
Se 77	108.8 ± 1.02	0.0075 ± 0.0015	0.1026 ± 0.013	0.367 ± 0.028	0.0037 ± 0.00051 *	0.0025 ± 0.00015	8 weeks
			3.8 ± 0.516*	0.93 ± 0.152	0.0049 ± 0.00071*	0.0043 ± 0.00075	12weeks
			2.8 ± 0.17*	0.7 ± 0.068	0.5986 ± 0.851*	0.145 ± 0.0135	16 weeks
			0.71 ± 0.075*	0.85 ± 0.121	0.2825 ± 0.0335*	0.1542 ± 0.0125	20 weeks
Sr 88	515.95 ± 5.1	19.575 ± 1.87	2.475 ± 0.4	2.57 ± 0.19	3.63 ± 0.46*	2.59 ± 0.35	8 weeks
			5.625 ± 0.41	13.43 ± 0.97	4.04 ± 0.27*	2.41 ± 0.25	12weeks
			12.58 ± 0.75*	3.34 ± 0.28	4.21 ± 0.65*	3.31 ± 0.24	16 weeks
			8.45 ± 1.51*	5.39 ± 0.23	3.76 ± 0.51*	3.46 ± 0.45	20 weeks
Ba 137	435.05 ± 7.95	8.83 ± 0.68	0.625 ± 0.09	0.68 ± 0.13	0.525 ± 0.048*	0.211 ± 0.032	8 weeks
			1.53 ± 0.159	3.17 ± 0.57	0.952 ± 0.11*	0.875 ± 0.102	12weeks
			7.62 ± 0.73	3.88 ± 0.48	1.02 ± 0.101*	0.737 ± 0.11	16 weeks
			3.48 ± 0.329	3.18 ± 0.47	0.94 ± 0.13*	0.85 ± 0.15	20 weeks
Pb 208	426.17 ± 4.97	0.0072 ± 0.0011	2.89 ± 0.298*	1.66 ± 0.21	0.18 ± 0.0157*	0.012 ± 0.00729	8 weeks
			3.617 ± 0.349*	1.46 ± 0.05	0.291 ± 0.0028*	0.129 ± 0.0255	12weeks
			8.95 ± 0.89*	3.55 ± 0.49	0.397 ± 0.026*	0.273 ± 0.013	16 weeks
			12.95 ± 0.49*	7.533 ± 1.059	2.11 ± 0.305*	0.3654 ± 0.0512	20 weeks

**Table 2:** Operation parameters for LA-ICP-MS.

Parameters	Values
RF power	1550 W
Sampling depth	5 mm
Sample (argon) flow	0.85 L/min
Isotopes measured	^12^C, ^13^C, ^23^Na, ^29^Si, ^31^P, ^39^K, ^43^Ca, ^47^Ti, ^56^Fe, ^59^Co, ^66^Zn, ^77^Se, ^88^Sr, ^137^Ba, ^208^Pb
Dwell time per isotope	0.02 s

## Abbreviations

ALP	Alkaline phosphatase
µCT	micro computed tomography
DEXA	dual-energy X-ray absorptiometry
LA-ICP-MS	laser ablation inductively coupled plasma mass spectrometry
OP	osteoporosis
OPG	osteoprotegerin
Ovx	ovariectomy
RANKL	receptor activator of nuclear factor kappa B ligand
TRAP	tartrate-resistant acid phosphatase
